# Attractor-Specific and Common Expression Values in Random Boolean Network Models (with a Preliminary Look at Single-Cell Data)

**DOI:** 10.3390/e24030311

**Published:** 2022-02-22

**Authors:** Marco Villani, Gianluca D’Addese, Stuart A. Kauffman, Roberto Serra

**Affiliations:** 1 Department of Physics, Informatics and Mathematics, Modena and Reggio Emilia University, 41125 Modena, Italy; gianluca.daddese.phd@gmail.com (G.D.); rserra@unimore.it (R.S.); 2 European Centre for Living Technology, 30123 Venice, Italy; 3 Institute for Systems Biology, Seattle, WA 98109, USA; stukauffman@gmail.com; 4 Institute of Advanced Studies, University of Amsterdam, 1012 GC Amsterdam, The Netherlands

**Keywords:** Random Boolean Networks, gene regulatory networks, critical systems, criticality principle, attractors, single-cell data

## Abstract

Random Boolean Networks (RBNs for short) are strongly simplified models of gene regulatory networks (GRNs), which have also been widely studied as abstract models of complex systems and have been used to simulate different phenomena. We define the “common sea” (CS) as the set of nodes that take the same value in all the attractors of a given network realization, and the “specific part” (SP) as the set of all the other nodes, and we study their properties in different ensembles, generated with different parameter values. Both the CS and of the SP can be composed of one or more weakly connected components, which are emergent intermediate-level structures. We show that the study of these sets provides very important information about the behavior of the model. The distribution of distances between attractors is also examined. Moreover, we show how the notion of a “common sea” of genes can be used to analyze data from single-cell experiments.

## 1. Introduction

Random Boolean Networks (RBNs for short) are strongly simplified models of gene regulatory networks (GRNs), proposed by one of us (Kauffman) more than 50 years ago, which have also been widely studied as abstract models of complex systems, thanks to the fact that their dynamical behavior can be tuned from ordered to disordered by modifying a few key parameters. They have also been applied to different biological phenomena, such as, e.g., cell differentiation [1,2,3], as well as to different fields, including robotics [4,5,6], the study of evolutionary processes [7,8,9] and the simulation of social systems [10,11,12,13].

It is possible to compare the behavior of RBNs to that of real biological GRNs, and thus, to draw inferences about their dynamics. The dynamics of RBNs is dissipative, so finite networks tend, after a transient has died out, to a limited number of different attractor states, which are either constant (fixed points) or periodic (limit cycles). It has been hypothesized that different attractors, which are persisting states where different nodes can take mutually coherent activation values, correspond to different cell types in multicellular organisms. On the basis of this correspondence, interesting scaling relationships regarding the number of genes and the number of different cell types or the duration of the cell cycle have been proposed, thus lending support to the hypothesis that ensembles of *critical* or *slightly ordered* (see below) RBNs can be useful to simulate the behavior of real gene networks [14,15,16]. This hypothesis has been strengthened by works showing that they can reproduce available data on HeLa cells [17] and on the distribution of avalanches of gene perturbations in yeast [18,19,20].

Both the topology and the transition functions of RBNs are indeed random, as described in Section 2.1, so they are well-suited to identify generic properties of these systems. At the time of their invention [21], very few detailed gene regulatory circuits were known (e.g., the lac-operon studied by Monod and Jacob), so the idea behind the use of random nets was that of exploring the possible outcomes of a web of interacting regulatory elements, without aiming at precise descriptions of specific circuits or organisms. This is why the study of specific networks was not pursued in those times, in favor of the search for the generic properties of statistical ensembles of networks. In a nutshell, the idea is that even evolved networks might share some of these generic properties.

It is, however, possible to use the Boolean framework to describe a specific gene regulatory circuit, if sufficient information is available, see, e.g., [22,23,24,25,26,27,28,29], although in this case the connections and the dynamical laws are no longer random, as they are based on available knowledge about how some genes affect some other ones.

Nowadays, the progress of genomic and related technologies makes it possible to guess the structure of several circuits. However, the number of well-known cases is still limited, and there are important uncertainties concerning the larger networks in which these approximately known circuits are embedded. Therefore, the search for generic properties using random networks is still an important research topic.

In RBNs, it is possible to identify regions of parameter space such that the typical dynamical behavior of a network belonging to those regions is either ordered (e.g., fixed points or periodic orbits) or disordered (e.g., pseudo-chaotic). Note that these remarks regard typical behaviors, while those of single network realizations (which are largely random) may of course be widely different from the typical ones. It was previously suggested that the separatrices between ordered and disordered regions may be particularly interesting, since networks whose parameters fall in those regions might be endowed with peculiar properties [14]. We will refer to those regions and networks as “dynamically critical regions (networks)”, or simply as “critical regions (networks)”.

It has been conjectured that critical networks should, at the same time, be robust and able to adapt to environmental changes. In changing environments, they should thus have an advantage over ordered or disordered nets; therefore, evolution should have driven biological networks to those regions [7,8]. This idea has been called the Criticality Principle [14,30,31,32], and it is the topic of this Special Issue. It has been suggested that the same reasoning applies also to networks that are ordered, but that have positions, in parameter space, that close to a critical region [14,20,33]. It can be noted that this hypothesis can also be extended to non-biological systems, as discussed in [6,9,32,34].

Because of their importance, critical networks have received a close examination, and have been the subject of several papers. Particular attention has been given to the number of attractors, to their scaling with the number of nodes, and to their dependence upon some key model parameters, such as, e.g., the fraction of so-called canalizing nodes [35,36,37,38]. However, there are also some important features that have remained largely neglected: this paper is dedicated to exploring the existence and the properties of a relatively large set of nodes that take the same value in all the attractors, a set that will here be called the “common sea” (CS). The nodes that do not belong to the common sea will be called specific nodes, as they take different “specific” values in different attractors, and their set will be called the “specific part” (SP).

As it has been observed, the study of RBNs is actually a study of families of different network realizations generated with the same parameters; therefore, it is necessary to clearly specify what the CS is. As described in detail in Section 2, RBNs are generated at random, either by drawing connections or by choosing Boolean functions, according to some probability distribution. The behavior of a specific network realization (defined by the set of connections and of the associated Boolean functions) might largely depend upon some idiosyncrasies of that realization, but the typical behavior of a large family of networks does not. For example, we might be interested in knowing how the number of attractors is affected by the choice of a given subset of the possible Boolean functions: in this case, we should study the dynamical behavior of different realizations, which all share the limitation to that subset. First, for each realization, it will be necessary to study the number of different attractors. Second, different realizations will be considered, and the appropriate statistical measures (e.g., average, median, standard deviation, etc.) will be taken. In this way, we will be able to associate a specific number (e.g., the average number of attractors) to a set of parameter values, when the analysis is limited to that subset of Boolean functions. The final step of the analysis might be that of studying, e.g., the way in which, in this case, the number of attractors depends upon the number of nodes.

Attractor states of RBNs are either constant (fixed points) or periodic (cycles) in time. However, for reasons discussed in Section 2.3, it makes sense to always make use of Boolean constant vectors instead of oscillating ones. Therefore, we define, for each dynamical attractor, a (constant) pseudo-attractor, whose components are identical to the (binarized) time averages of the corresponding components of the true attractor. How this is achieved is precisely described in Section 2.3. The mapping from true dynamical attractors to pseudo-attractors is not injective, so in Appendix B, we provide an estimate of how it affects the number of different asymptotic states (i.e., number of pseudo-attractors vs. number of true dynamical attractors.)

We define the common sea as the set of nodes that take the same values in all the pseudo-attractors of a given network realization. By studying different realizations, with different parameter values, we will be able to understand how the CS depends upon the features of the network. We will study the properties of the common sea on different networks generated using the same set of parameters (in particular, the connectivity *k* and the bias *b*, defined below in Section 2). Then, we will compare these properties in families of networks with different parameters, with a particular focus on critical networks.

Note that the concept of “common sea” is a new one, and that it differs from previous definitions. Fixed points are sometimes referred to as “frozen nodes” in the literature on RBNs [14], and whole portions of the network are sometimes called “frozen sea” [15]. The definition of the common sea is different, since (i) it is based on pseudo-attractors, so that also nodes that oscillate in the “true” dynamical attractor may belong to the CS and (ii) it requires that the nodes take the same value in all the attractors, so that some nodes that are “frozen” in some attractors may not belong to the CS. Property (i) distinguishes the common sea also from the “stable cores” defined in [39,40]

It is also interesting to observe the internal organization of the common sea and of the specific part. In the cases that are described in this paper, the overall network is not made by disjoint subparts: although this is not prohibited, it is extremely unlikely within the range of parameters that are explored. However, once the CS of a given network realization is identified, we can look at the topology of the network that is composed of its nodes only. For reasons detailed in Section 2, we identify the subparts of the CS with its so-called weakly connected components (WCCs) [41] and we refer to the whole process as “fragmentation”.

As it will be detailed in Section 3, in the CS there is sometimes only one WCC per network realization, but in other cases, there are more than one, although one usually finds a dominant subpart that comprises many more nodes than the others. Of course, a similar fragmentation analysis can be conducted on the specific part, and here we often find a more evenly distributed situation, with more fragments of more similar size. It is natural to refer to these WCCs as “islands”, and to the specific parts as “specific islands”. It should be emphasized that these are not completely independent parts, and that some changes in one WCC (for example, the knock-out of a gene) can affect the values of nodes in other WCCs.

Another important feature is the distribution of distances between pairs of pseudo-attractors, the natural choice being their Hamming distance. Note that distances are limited from above by the size of the common sea, which, for a quite large set of parameter values, comprises a large fraction of the network nodes. The distribution of distances in different ensembles of Boolean networks will also be presented in Section 3.

While this work deals mainly with the properties of dynamical models (i.e., in-silico networks), the motivations for interest in RBNs are deeply rooted in biology, so we will also briefly discuss some experimental results about the biological analogue of the common sea and of the distribution of distances, which are made possible by the available technology, identifying the model pseudo-attractors with observed cell types.

We stress that we do not aim to perform a careful comparison of the model behavior with experimental data, which would require, inter alia, a more thorough analysis of the effects of binarization, an analysis of the behavior of RBNs with different parameter values and maybe a larger data set. Indeed, we do not provide in the paper any direct quantitative comparison of model with data. Our goal is rather that of showing that the study of an abstract RBN model brings us to look at experimental single-cell data with an interesting system-level perspective, which differs from the more specific questions that are usually addressed. To the best of our knowledge, no study of the “common sea” and of the distance distribution has been proposed and single-cell data have been mainly used to identify genes with expression levels that are associated with specific cell types, or for network reconstruction ((different types of) distances between pairs of cell types are often computed, e.g., for the purpose of clustering [42] or looking for the number of differentially expressed genes [43], but we are not aware of studies that are specifically devoted to the distributions of gene distances). We had already encountered a similar situation in our previous works on avalanches of gene expression changes in S. Cerevisiae [18,19,20], where it was also possible to use data, which had been collected to address specific biological questions, to answer interesting generic, system-level questions.

In the experiments we consider here, the expression levels of many genes in a single cell are measured. These data are very noisy; therefore, it is necessary to sum the contributions of several instances (of the same type) to obtain a reliable average profile for a given cell type. In this way, one obtains a set of values that might be compared with the components of the “average attractor” mentioned above, taking into account, however, that the latter are restricted to the interval [0,1] while the former are not. The biological literature refers to these typical expression profiles as pseudo-cells [44,45], which should, of course, not be confused with the pseudo-attractors of this paper, which are defined for simulation models only. In order to compare them with Boolean pseudo-attractors, it is necessary to cast real-valued experimental data on pseudo-cells in Boolean form. This can be achieved by defining a threshold, so that a gene can be considered active if and only if its activation exceeds the threshold. In this way, we can define Boolean pseudo-cell types by associating to each gene the value 0 if its activation value is smaller than the threshold, and the value 1 otherwise. A large, important set of human single-cell data will be analyzed in this way, and the results will be presented in Section 4.

Section 2 of the paper is organized as follows.

1. A synthetic description of the RBN model, which makes it possible to read the paper without any a priori knowledge of RBNs (without, of course, covering all their main properties). Since some slightly different versions of RBNs have been proposed, it is also precisely stated which one is used in this paper;
2. The main method to evaluate whether Boolean networks are dynamically ordered, critical or disordered (Derrida parameter);
3. The definitions of average attractor, pseudo-attractor, common sea, specific part;
4. The methods used to analyze the common and specific parts;
5. The methods used to collect and to represent single-cell data, obtained from the literature.

In Section 3, the common sea will be studied through simulations, emphasizing how its size depends upon the main system parameters. We will deal mostly with dynamical critical networks, but we will also consider some cases of ordered networks, for the sake of comparison. A simplified, mean-field analytical estimate of the size of the common sea will also be provided in Appendix A. We will consider networks with different values of the number of inputs per node, *k*, and of the bias *b* of the Boolean functions, which is defined in Section 2 as the average value of the number of outputs equal to “1” in their truth table, and we will see how the size of the CS depends upon these two parameters. They may be varied independently, but the requirement that the network be critical (i.e., that the value of the Derrida parameter be 1) defines a relationship between them. Thus, there may be critical networks with different pairs of values of *k* and *b*. It will be interesting to observe that the size of the CS is not uniquely determined by the constraint of criticality. The distribution of distances between pairs of attractors will also be studied in Section 3.

Section 4 is dedicated to the analysis of experimental data, using Boolean pseudo-cell types as analogues of Boolean pseudo-attractors to identify the biological “common sea” and to study the distribution of distances. Boolean pseudo-cell types are obtained from real-valued data, and they heavily depend upon the choice of the threshold. In single-cell experiments, one often finds a large fraction of null values, so a possible choice is that of regarding as identical only those genes that take exactly the same activation values in each cell type: this corresponds to adopting a vanishing threshold. It is, however, unlikely that nonzero activation values that are identical will be found under this quite severe requirement. Moreover, the high noise level makes it appropriate to consider nonvanishing threshold values. Of course, if we adopt a very high threshold, all the nodes will turn out to be unexpressed. There is no firm a-priori reason to fix a particular value of the threshold, but we will suggest some possible heuristic criteria in Section 4.

Several results will be presented in Section 3 and Section 4, so in the final part, Section 5, we will summarize those that seem, to us, to be the most relevant and stress their importance. We will also indicate promising directions for future work, distinguishing some improvements that are quite straightforward from other improvements that require more care.

## 2. Materials and Methods

As anticipated in the introductory Section 1, we will here describe both methods and definitions that are relevant to this work. The chosen methods are quite well-known, while some definitions are original.

### 2.1. Random Boolean Networks

RBNs have been very popular, and they are addressed in many papers. We will not provide a complete bibliography, but we will rather refer the reader to some original or review works [14,16,40,46]. Several variants of the original model have been introduced and it is, therefore, necessary to precisely define which one is used here. A RBN is a time-discrete, directed network of N nodes, or genes, which can take one of two possible values, sometimes called activation values or states of the node (we will use 0 and 1 in the following). Each node has a fixed set of input nodes (or input links) and a fixed set of output nodes: information flows from the input nodes to our node, and from our node to its output ones. We will consider only the so-called quenched model, which is the only one that makes sense if one thinks of a finite gene regulatory network. The alternative annealed model may be a useful approximation that allows one to perform some analytical calculations in the case of very large networks (N → ∞) [40,47]. A fixed Boolean function is associated with each node, which determines the value of that node at time *t* + 1 from the values of its input nodes at the previous time step *t* (self-connections being prohibited). In the simulations of this paper, the updating is synchronous, i.e., all nodes’ values are updated at each time step.

The nodes are numbered from 1 to *N*, and a state of the network is a *N*-dimensional Boolean vector ***X*** = (*x*_1_…*x_N_*), whose components are the activation values of the corresponding genes. The set of Boolean functions defines a mapping ***X***(*t*) → ***X***(*t* + 1), i.e., a time-discrete dynamical system.

***X***(*t* + 1) = F(***X***(*t*)), (1)

Note that, while the network is built in a random way (detailed below), the dynamics of a single network are deterministic, i.e., the state at time *t* determines the state at time *t* + 1. It is easy to see that a finite network always tends in a finite number of steps to a constant state, or to a cycle (i.e., a state where ***X***(*t + T*) = ***X***(*t*) for some finite *T*, the period of the cycle). Fixed points are cycles with *T* = 1.

Since asymptotic states are cycles of finite length, so are the possible attractors of the network. The basin of attraction of an attractor is the set of states that tend towards that attractor under the mapping (1).

Both connections and Boolean functions are chosen at random in RBNs, although sometimes with different approaches. We will follow Kauffman’s original proposal, thus assuming that all the nodes have the same number k of incoming links, coming from the other nodes, chosen at random with uniform probability. In the case of large sparse networks, this procedure gives rise to an approximately Poissonian distribution of outgoing connectivities. Of course, other alternatives are possible, but the “classical” model has proven able to describe some interesting features.

The Boolean functions are also chosen at random for each node, independently of those of the other nodes. They are the transition functions of the model, i.e., they determine the state at time *t* + 1 of the node, on the basis of the values of its inputs at time *t*. Sometimes they are chosen with uniform probability in a set of functions, identified because of some peculiar features or because they appear to be biologically plausible. Another procedure, followed in this work, is that of assigning at random, for each possible set of input values, an output value equal to 1 with probability *b*, which is called the bias of that set of Boolean functions (the probability of assigning 0 being, of course, 1 − *b*).

### 2.2. Determining the Dynamical Regime

As previously observed, the dynamical behavior is particularly important. Ordered and disordered dynamical regimes are usually described as systems’ behaviors supporting, respectively, short attractors with fairly regular basins of attraction and long attractors with sensitive dependence upon initial conditions (this is why disordered states are often called “chaotic” even if in RBNs the time step and the values of the variables are discrete). In ordered networks, a change at one node (i.e., a bit flip) propagates in one step, on average, to less than one other node: starting from a random initial condition, therefore, an ordered system rapidly reaches a stable condition where the majority of nodes are frozen (i.e., constant in time); if this asymptotic behavior is perturbed, there is a very high probability of coming back to it. On the contrary, in disordered networks, a perturbation at one node propagates in one time step, on average, to more than one other node: very close initial conditions could rapidly diverge toward different attractors, and attractors typically have long periods, with large portions of oscillating nodes. If perturbed, the system therefore has a high probability of changing its asymptotic behavior. Critical systems are at the boundary between these two dynamical regimes, where a change at one node propagates in one time step on an average to exactly one other node.

There are two main different ways to identify ordered and disordered regions: (i) a static one, based upon the knowledge of the nodes average connectivity and the bias of the Boolean functions [48] and (ii) a dynamical one based upon the actual study of the spreading of perturbations through the system [14,47,49]. Here, we will use only this latter approach (note that the two provide the same results if the system is ergodic [50]).

The approach considers the short time evolution of the distance between two initial states that differ by a small fraction of nodes. At each time step, the difference between the states of the two runs is measured by means of the Hamming distance *h*(*t*), the number of nodes that have different activations. If the network is operating in the disordered regime, then small Hamming distances tend to diverge and the ratio *h*(*t* + 1)/*h*(*t*) is likely to increase; on the other hand, networks in the ordered regime exhibit convergence for nearby states, so *h*(*t* + 1)/*h*(*t*) tends to decrease. Critical states separate those cases, *h*(*t* + 1)/*h*(*t*) tends to remain the same. In the search for generic properties, one should, as usual, average over many pairs of initial states and over ensemble of random networks. Denoting these averages by square brackets, one can now define the Derrida parameter *λ* as follows:

(2) $$\lambda \equiv \frac{\langle h\left(t+1\right)\rangle }{\langle h\left(t\right)\rangle }\;\;\;\;\;\;if\;h\left(t\right)\;\to \;1$$

The *λ* parameter can be regarded as a discrete analogue of the Lyapunov exponent of continuous dynamical systems: the network is ordered if *λ* < 1, disordered if *λ* > 1 and critical if *λ* = 1.

Several previous studies have focused on the identification of the parameters that are responsible of the various dynamical behaviors, and on the features of the system’s phase space (attractors and basins of attraction). It has been shown that, for ensembles of networks generated in the way described above, there is a relationship between the number of connections per node and the bias that characterizes critical states, i.e.,

(3) $$k=\frac{1}{2b\left(1-b\right)}$$

For example, networks with *k* = 2 and *b* = ½2 are critical.

### 2.3. Definitions of Average Attractor, Pseudo-Attractor, Common Sea and Specific Part

The identification of which genes “take the same value” in different cyclic attractors requires some care, since cycles in RBNs depend to a large extent upon the choice of synchronous updating, which does not have a sound biological basis [46]. Synchronous RBNs are Markovian systems, whose state ***X***(*t* + 1) depends upon ***X***(*t*), forgetting the previous states of all the nodes of the network. The action of a gene on the activation of other genes takes place through the action of its corresponding protein; therefore, the notion of a single time step corresponds to assuming a common decay time of the different regulatory proteins, which is not supported by biological data. In previous papers [51,52,53], we proposed a different model, the gene–protein model, where a distribution of decay times is introduced. However, this more complicated model would require further parameters: in this paper, we therefore focus on the much better known “classical” RBNs. Indeed, cycles are not robust with respect to, e.g., small perturbations [54] or to asynchronous updating [55], which is, however, also unrealistic.

Moreover, with a view to a possible comparison with experimental data that are not time dependent, it is advisable to resort to an approximate description of the dynamical attractors’ constant “pseudo-attractors”, precisely defined as follows.

Let ***A*** be a (possibly time dependent) dynamical attractor, let *A_k_* be its *k*-th component and, if *A_k_* changes in time, let *f_k_*_1_ ∈ [0,1] be the ratio between (the number of states in the cycle where *A_k_* = 1), and the overall period *T* of the cycle. Let also *θ* a real number in [0,1]. To every dynamical attractor ***A***, we associate its “pseudo-attractor” ***A***′, whose component *A*′*_k_* takes the value

1. 1, if *A_k_* either takes the constant value 1 or if *A_k_* oscillates and the corresponding *f_k_*_1_ > *θ*;
2. 0, in other cases.

Obviously, constant states in ***A*** are mapped onto the same states in ***A***′, while oscillating states are mapped onto the constant state 1 if their time average exceeds the threshold *θ*, and onto the constant state 0 if the average is equal to or smaller than the threshold.

In the following, we will often use the term attractor to refer also to pseudo-attractors (when necessary, we will call them precisely pseudo-attractors). The introduction of pseudo-attractors represents a kind of coarse graining, which maps different true dynamical attractors onto the same pseudo-attractor; a simulation-based estimate of the loss of detailed information that is associated to this coarse graining is provided in Appendix B.

The common sea (CS) is defined in Section 1 as the set of nodes that take equal values in all the pseudo-attractors of a given network realization, which, in turn, is defined by its topology (which genes are connected to which other genes) and by its Boolean functions (which are generated once and for all for each realization). By varying the initial conditions, different attractors can be discovered. Note that, for a network of *N* nodes, there are 2*^N^* possible initial conditions, a number that quickly becomes huge—so the actual search of attractors is necessarily bound to explore only a subset of all the possible initial conditions. This is, however, a well-known problem in the study of RBNs (except in the case of very small networks) and it affects the study of all their important properties. Extensive simulations (in our work we used 10,000 initial conditions for each network realization), however, allow the identification of those attractors that have a significant basin of attraction. It may be argued that attractors with very small attraction basins, which may be overlooked in this way, are also unlikely to play any significant role in biological systems.

The specific part (SP) is the set of all the nodes that do not belong to the CS. Note that, since the SP is often composed of relatively small subparts, they are often called the “specific islands” (to match the use of the term “sea” for the common part).

### 2.4. Methods to Analyze the Fragmentation of the CS and of the SP

As anticipated in Section 1, using the “lens” of pseudo-attractors, it is possible to identify meaningful subparts in the common and specific parts of RBNs. This does not happen with an appreciable probability in the case of the whole network, with the parameter values used in this study. The fragmentation of the CS and of the SP can be extremely interesting since it might highlight intermediate-level structures that spontaneously form in networks that are otherwise fully random.

**Figure 1:**
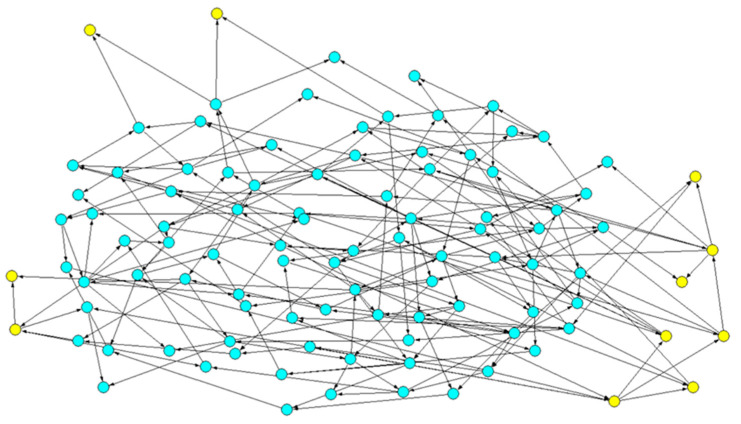
Example of a particular topology: the common sea is in blue; the specific part is in yellow. The two sets form two directed graphs: it is, however, possible to ignore the direction of the links, and thus identify the number of subgraphs in which the common part and the specific part are fragmented. This operation corresponds to the search for the weak connected components in the respective subgraphs: in the figure, we highlight the four WCCs in which the specific part is fragmented. It can be observed that a WCC could be composed of a single node (in the upper part of the figure), with both upstream nodes belonging to the common part: this situation is not unusual, as a large part of the common part is composed of oscillating nodes (see the following part of this section).

To analyze the fragmentation of the CS or of the SP (let us refer to the latter to simplify the writing), we first identify it, from its definition, and we highlight its nodes. In the example of Figure 1 below, its nodes are yellow, while those of the CS are blue. We look at the links between the nodes in the SP and, ignoring the direction of the arrows, we identify its Weakly Connected Components. Let us recall that a directed graph is weakly connected if, replacing its edges with undirected edges, a connected graph is obtained, i.e., one where there is a path between any pair of vertices. A WCC is a maximal connected subgraph of the undirected graph.

Note that the subparts thus identified are not totally independent of each other. For example, by knocking out (i.e., permanently silencing) a gene in a WCC of the SP, the values of genes in the CS or in other WCCs can be modified. For example, 48% of the knock-outs (KOs) applied to the largest WCC of the specific part in Figure 1 reach one or more of the other WCCs of the specific part, while no KO applied in these WCCs is able to reach the other WCCs. The avalanches were computed following the method presented in [18,19,20]. However, the notion of a WCC captures the idea of some form of modularity, in that the interactions within the component play a key role in determining its behavior. This is likely to be true when the WCCs have similar sizes, while if one component is much larger than the others, it may have a strong effect.

### 2.5. Methods to Represent Single-Cell Data

As already observed, we used, in this study, data from the literature, without directly performing laboratory experiments. There are several gene expression databases available in the literature. However, it is difficult to find sufficiently pure genetic profiles: until recent years, in fact, the profiles of cell types derived from bulk type analyses, in which many different cells are mixed in the sample, without the possibility of knowing the exact composition of the cell types under scrutiny. On the other hand, in the last few years, databases of expression of single cells (SC in the following) have flourished, giving rise to “atlases” that store the data of a huge number of SCs. Thus, it is now possible to profile thousands of single cells of the same type [56,57], giving rise to a cell atlas of whole organisms [45,58,59].

A very useful atlas for our purposes is described in [45], concerning a comprehensive cell landscape for humans. Single cells were processed using microwell-seq16 and sequenced at around 3000 reads per cell. The dataset contain 599,926 different single cells. The data include samples of both fetal and adult tissue and cover 63 human tissue types. As described in [45], further analyses make it possible to further refine the classification, leading to the identification of 102 different clusters that can be mapped to the starting tissues, allowing interesting observations. In this work, however, we were limited to using the initial classification of 63 types, performed by human experts.

Single cell data are very noisy and, moreover, the activities of single cells are influenced by their own phase of functioning and by the surrounding environment. However, since many different exemplars of each type are available, it is possible to aggregate all the contributions into a single profile that then constitutes the “average profile” of the cell type to which the single cells refer. For this purpose, we used the data from [45], after having normalized them using the DESeq2 [60] method. These “pseudo-cells”, therefore, have some similarities with the average attractors of Section 2.3, although in the case of biological data, they are not bound to stay within the range [0,1].

In order to compare experimental data with simulation results, it is necessary to cast the data on pseudo-cells in binary form, thus giving rise to a binary pseudo-cell type. This can be achieved by introducing a threshold *χ*, according to one of two possible approaches. In the simplest one, the real-valued components of the pseudo-cell type are compared with a fixed threshold (the same for all the genes of all cell types). However, since the maximum expression values of different genes may be very different, the use of a single threshold is questionable. In the second type of approach, each gene expression level is re-scaled as a fraction of the maximum observed value of that gene in all cell types.

The two approaches can be formalized as follows:

**Approach 1**: Let ***C*** be the vector of the pseudo-cell, whose real-valued components *C_k_* are the aggregate levels of expression of the various genes. Then, the Boolean pseudo cell type is represented by the vector ***D*** whose components:

1. Take the value 0, if *C_k_* < *χ*;
2. Take the value 1, if *C_k_* ≥ *χ*.

**Approach 2**: Let *M_k_* be the maximum observed value of the *k*-th gene, and let ***C***′ be the vector obtained by ***C*** by dividing each component by the corresponding *M_k_* (*C*′*_k_* = *C_k_*/*M_k_*).

Then, the Boolean pseudo cell type is represented by the vector ***D***’ whose components:

1. Take the value 0, if *C′_k_* < *χ*;
2. Take the value 1, if *C′_k_* ≥ *χ*

## 3. Results of Simulation Models

As anticipated, in this section, we will study the common sea and the specific part of Random Boolean Network models, using computer simulations. Unless otherwise stated, we will report results of simulations of ensembles composed of 1000 networks with *N* = 100 nodes and *k* = 2 inputs per node, each network evolving from 10,000 random initial states generated by assigning, at random, a value 0 or 1 to each node with independent identical probabilities (equal to ½). The simulations continue until an attractor state is reached; the attractor is used to determine the corresponding pseudo-attractor. The CS and the SP, as well as their WCCs, are identified according to the methods described in Section 2. The distances between pseudo-attractors are Hamming distances.

We first consider the properties of the most widely studied case, i.e., the case of critical networks with *k* = 2, *b* = ½ (Section 3.1). Later, in Section 3.2 we consider also ordered networks with higher values of the bias, and in Section 3.3 we examine critical networks with different pairs of values of *k* and *b*. In the last part of this section, we look at the distribution of distances between pairs of pseudo-attractors.

### 3.1. Common Sea and Specific Part in Ensembles of Critical Networks with Bias = 0.5

**Figure 2:**
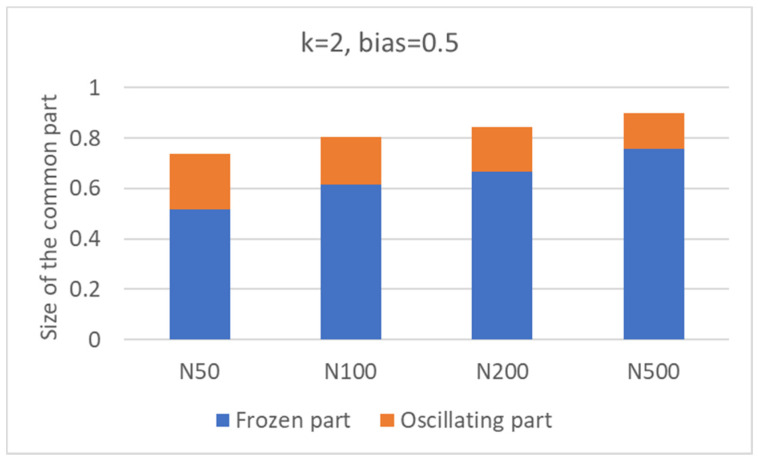
Size of the common part (fraction of the total size of the network), as the number of nodes increases. Inside the columns, the fractions occupied by the frozen part and the oscillating part are visible (see later in the text).

A first observation concerns the scaling of the size of the CS with that of the whole network. In Figure 2 (which is not limited to nets with *N* = 100), one can see that the relative size of the common sea increases as the number of nodes of the network is increased.

A perhaps surprising observation concerns the large size of the CS. It is possible to show with a simplified mean-field calculation that this is a property of RBNs with *k* = 2, *b* = ½. Indeed, in this case, all the 16 two-input Boolean functions are equally probable. So, in the limit *N* → ∞, we expect that *N*/16 nodes will always take the value 0, and *N*/16 will always take the value 1. Therefore, at least *N*/8 (12.5%) nodes are always constant. Furthermore, constant nodes tend to make some of their descendants also constant. An oversimplified calculation (sketched in Appendix A) shows that the actual fraction of constant nodes easily grows, thus justifying the large values of Figure 2, which were obtained by simulating finite networks.

**Figure 3:**
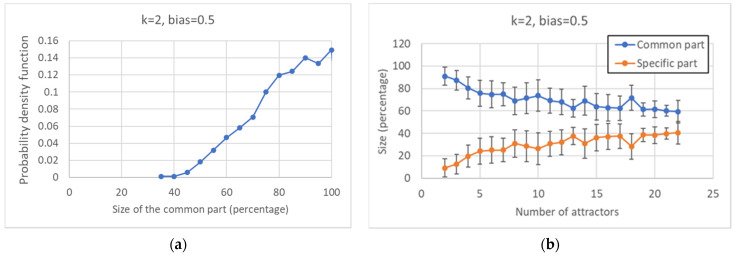
Characterization of the common and specific parts I. (**a**) Distribution of the size of the common part. (**b**) Average size of the common and specific part vs. the number of attractors: the error bar is three times the standard deviation of the mean, a situation that corresponds to a confidence interval of 99.7% in case of Gaussian distributions.

Looking at the distribution of the size of the common sea in ensembles of networks with a fixed number of nodes (100), one can observe that it tends to privilege higher values (Figure 3a), reaching its peak when the size of the CS equals that of the whole network; the average size exceeds 80% of the size of the RBN.

The size of the specific part is equal to the difference between the total number of nodes and the size of the common sea; therefore, it is a decreasing function of the latter. It is interesting to observe that the number of attractors found is also negatively correlated with the size of the common sea (Figure 3b).

**Figure 4:**
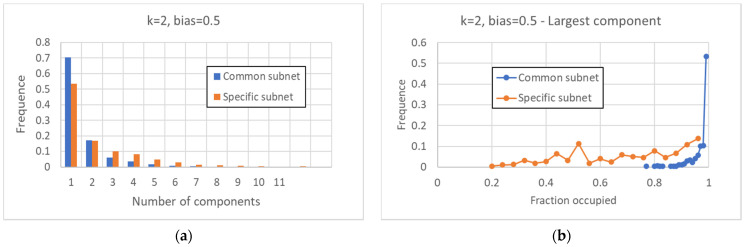
Characterization of the common and specific parts II. (**a**) Distribution of the number of the WCCs for the common part and the specific part. (**b**) Fraction on the total of the major fragment of the common (specific) part, in the case in which this part is divided into at least two WCCs. In more than 50% of cases, the greater fragment of the common part occupies more than 98% of this part; the larger fragment of the specific part can instead have much smaller values.

As far as fragmentation is concerned, one observes that the common sea is generally quite compact, and in 70% of the cases, the graph is composed of a single WCC; in the remaining 30% of cases, this part is divided into two or more WCCs (Figure 4a). The specific part is more fragmented than the common part: it has a single WCC in just over 50% of cases, and whenever it is fragmented into more than one component, the size of the largest WCC is relatively small (Figure 4a,b).

**Figure 5:**
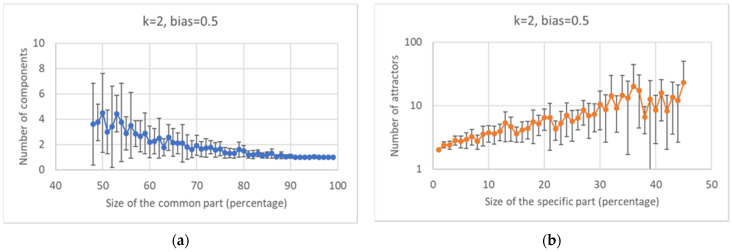
Characterization of the common and specific parts III. (**a**) Number of WCCs in the common part vs. the size of the common part (the same trend is present in the specific part—data not shown). (**b**) Number of attractors vs. size of the specific part.

In Figure 5a, it can be observed that the greater the size of the common part, the smaller the number of parts into which it is fragmented (and the smaller is the variance of the distribution). Another interesting observation is the one concerning the (slightly weak) positive correlation between the size of the specific part and the number of attractors (Figure 5b).

**Figure 6:**
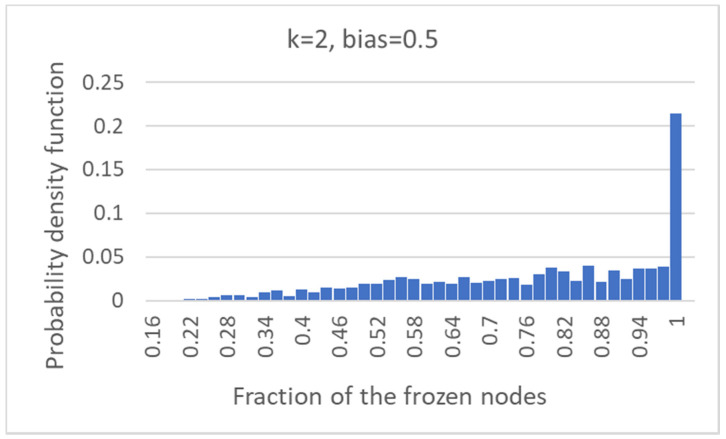
Frozen common sea: the distribution of the fraction of the common part that is composed of fixed (frozen) nodes.

Finally, it is possible to observe how many nodes of the common part are indeed frozen nodes (that is, non-oscillating nodes, with activation values constant at 0 or at 1 in the original dynamical attractors). In slightly more than 22% of cases, the common part is made up of all fixed nodes, but in about 78% of the cases, the common part comprises oscillating nodes (Figure 6).

### 3.2. Dynamically Ordered Networks

Let us now observe how the characteristics described above vary as dynamical regime of the networks is changed, keeping the average connectivity fixed to two input links per node, and modifying the bias. Note that choosing a bias different from ½ implies, in this case, that the dynamical regime is ordered. For simplicity, we will consider only the case of increasing bias, i.e., increasing the fraction of “1”s in the truth tables of the Boolean functions; the same phenomena would take place by increasing the fraction of “0”s.

**Figure 7:**
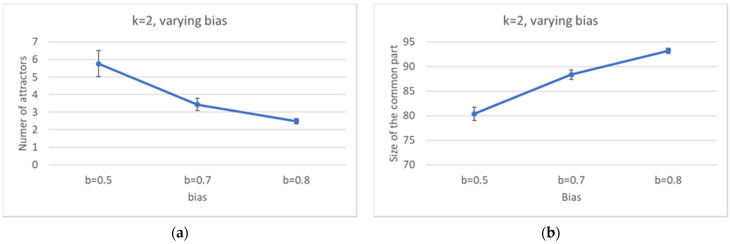

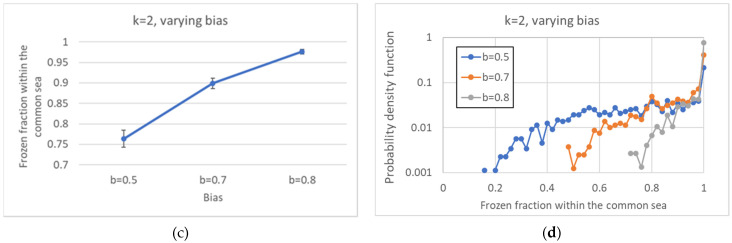
Increasingly ordered ensembles. (**a**) Average number of attractors. (**b**) Average size of the common part. (**c**) Average frozen fraction within the common part. (**d**) Distribution of the size of the frozen fraction within the common part (logarithmic scale). Note the sharp increase in both the minimum size of the frozen fraction, and the frequency of appearance of completely frozen common parts, as the bias increases.

**Figure 8:**
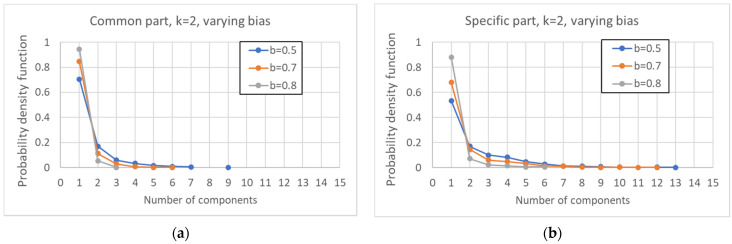
Distribution of the number of (weakly connected) components into which the common part (**a**) and the specific part of each network (**b**) is divided, as the bias varies.

By increasing the bias, the distribution of the number of pseudo-attractors also varies, leading to systems with a smaller average number of attractors (Figure 7a). At the same time, the common part grows in size (Figure 7b), it is composed by less WCCs (Figure 8), and it is composed of less and less oscillating nodes—Figure 7c,d.

In Section 3.1, it was observed that, the larger the CS, the less fragmented it is (and the same happens for the SP). The same happens to the ordered networks of this Section.

### 3.3. Critical Networks, with Different Connectivity and Bias

In this Section, we modify the connectivity *k* (i.e., the number of input connections per node) of the system, and we simultaneously change the bias in order to maintain the RBN dynamically critical, according to Equation (3). Criticality is also checked by verifying the Derrida parameter

**Figure 9:**
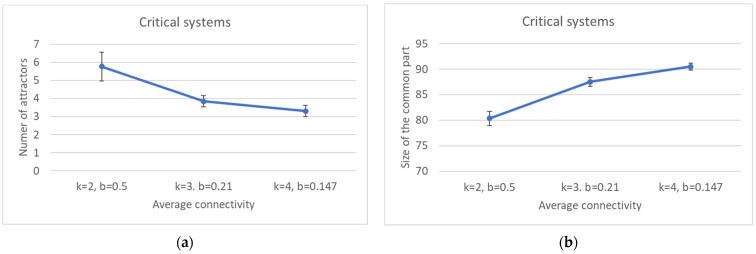
Critical systems with increasing connectivity: (**a**) number of attractors; (**b**) size of the common part.

By increasing *k*, one observes a decrease in the average number of attractors and an increase in the size of the common part (Figure 9a,b). A straightforward consequence is that the condition for criticality (Equation (3)) does not uniquely determine the size of the common part, which seems to be affected by either k or b, or some other combination of the two.

**Figure 10:**
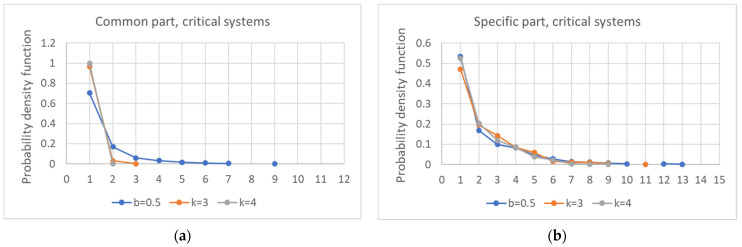
Distribution of the number of components into which the common part (**a**) and the specific part of each network (**b**) is divided, as the connectivity varies.

As previously observed, the size of the common part affects its fragmentation, which decreases as the connectivity increases (Figure 10a). The same phenomenon is also observable in the specific part, albeit in a weaker form (Figure 10b). Furthermore, the fraction of systems in which the specific part consists of a single component as the connectivity varies does not increase (data not shown).

The ensembles used so far are characterized by the number of nodes in the network *N*, the average connectivity *k* and the bias *b* of the Boolean functions. The number of nodes *N* has no effect on the dynamical regime—except for the fact that larger networks have smaller deviations from the dynamical regime of their ensemble.

A comment is in order. In the previous part, Section 3.2, we noticed that, in case of *k* = 2, the increase in the bias from its value corresponding to criticality to the ordered region leads to the increase in the size of the common part, the decrease in its fragmentation, the decrease in the number of oscillating nodes and the increase in the probability of being composed of only one WCC. Something similar takes place in this Section 3.3, but here the effects of the increase in the bias are to some extent counteracted by the corresponding increase in the connectivity (which would have led the network towards a chaotic regime, if the bias had not been modified).

### 3.4. Distribution of Distances

**Figure 11:**
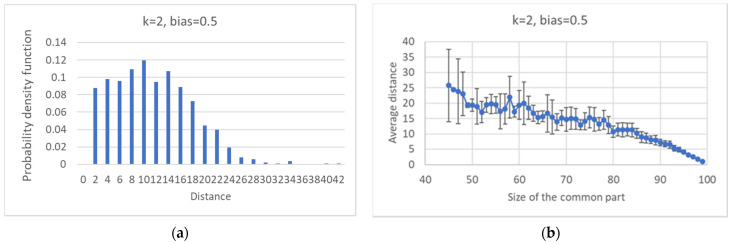
Critical ensemble with *k* = 2 and bias = 0.5. (**a**) Distribution of average distances between attractors (each RBN is contributing with the average of its distances). (**b**) Average distance vs. the size of the common part.

It is possible to compute the distribution of distances between pseudo-attractors belonging to the same ensemble, and to observe their dependence vs. the size of the common part (Figure 11).

**Figure 12:**
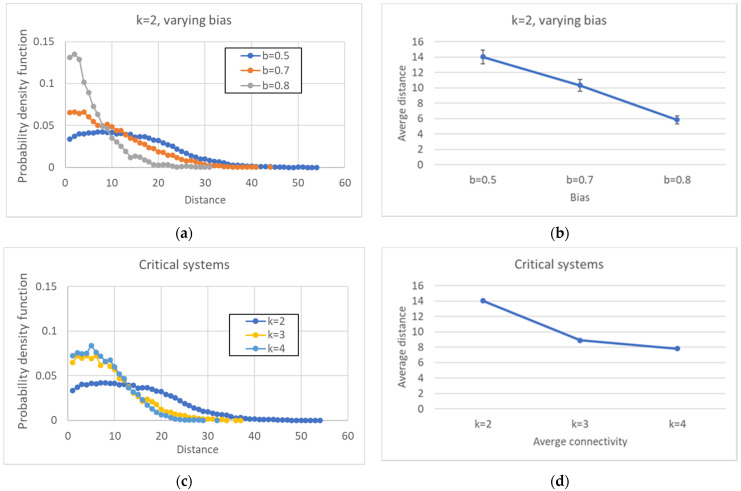
Distribution of all the distances between attractors belonging to the same ensemble. (**a**) *k* = 2, varying bias. (**b**) Average distance between attractors, varying bias. (**c**) Average distance between attractors, critical systems, varying connectivity. (**d**) Critical systems, varying connectivity.

In Figure 12, the distributions of all the distances are presented. Figure 12a,b refer to networks with fixed *k* and changing *b*, while Figure 12c,d refer to critical networks with different connectivities. These distributions are, of course, consistent with the phenomena observed in the previous sections since the distances between pairs of attractors are affected by the size of the common part.

**Figure 13:**
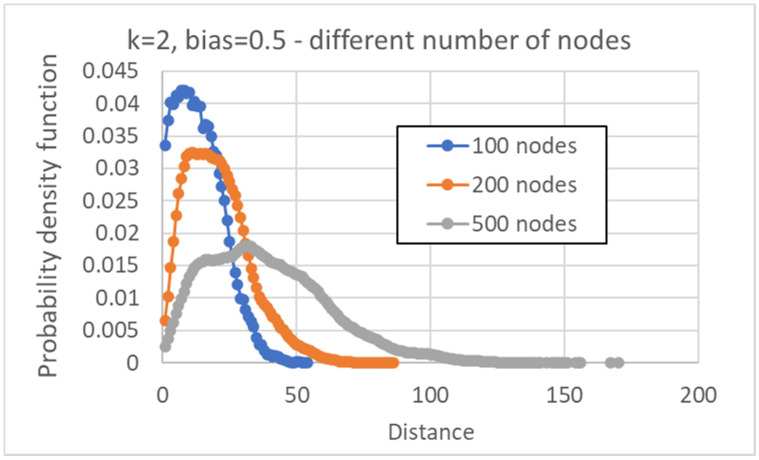
Distribution of distances between attractors belonging to the same network, *k* = 2, bias = 0.5, varying the number of nodes. The average distance varies from 14.0 ± 0.1 (RBN with 100 nodes), to 20.67 ± 0.04 (RBN with 200 nodes), to 39.08 ± 0.03 (RBN with 500 nodes.

In the following part, Section 4, we will present data about the distribution of distances between pairs of human cell types, obtained from single cell experiments. Those networks are made of 27,341 genes, while our simulations are limited to a much smaller number of nodes. To obtain a feeling for the effects of a larger number of nodes, we show below the distributions of distances between pseudo-attractors, in the case *N* = 100, 200 and 500 (keeping *k* = 2, *b* = ½). It can be observed that an increase in *N* tends to broaden the interval of distance values with an appreciable number of corresponding cases, and to shift the maximum of the distribution to the right, while lowering its height. In every case, the distribution is unimodal, with a single maximum at a nonvanishing distance value (Figure 13).

## 4. A Look at Experimental Data

The study of mathematical models allows the identification of interesting questions, which can provide useful insights and viewpoints concerning real genetic regulatory systems. In turn, the analysis of biological data can provide indications about the validity and the limitations of theoretical models, thus leading to a (hopefully fruitful) feedback between theory and simulation on one side, observation and experiments on the other. In this paper, we introduced, in the RBN model, the notions of common sea and specific part, and we examined their properties. It is now interesting to look at the applicability of these notions to biological data, keeping in mind that models are anyway the main focus of this paper.

Let us also preface that it would not make much sense to present here quantitative comparisons between the results of our simulations and biological data. The former were meant to uncover the main properties of these new concepts, and were based on extensive simulations of networks of limited size (in most cases, composed of 100 nodes), while the numbers of genes in biological multicellular organisms are much larger (27.341 in the case discussed in this section). However, we find that even a qualitative comparison can be useful and provide novel viewpoints useful both for models and for data analyses.

In order to apply the notions of CS and SP, it is necessary to associate our pseudo-attractors to biological entities. Although other alternatives are possible (see, e.g., [1,61,62]), here, we follow the original Kauffman hypothesis [14,15], which identifies attractors with typical long-lived states of a cell; that is, in multicellular organisms, with cell types.

It is important to make use of single-cell data, since bulk data may mix contributions from different types. As described in Section 2, we will refer to an important set of such data [45], concerning a comprehensive cell landscape for humans, which contains 599,926 different single cells and 27.341 genes. Since single cell data are very noisy, and often plagued by false negatives, we construct pseudo-cell types by averaging all the contributions into a single profile, and then binarizing them with a threshold, as described in the Methods section. These Boolean pseudo-cell types can then then be considered the biological analogues of the pseudo-attractors.

As described in Section 2, there are two alternative ways to binarize the data; the corresponding results are given here below

### 4.1. Approach 1

**Figure 14:**
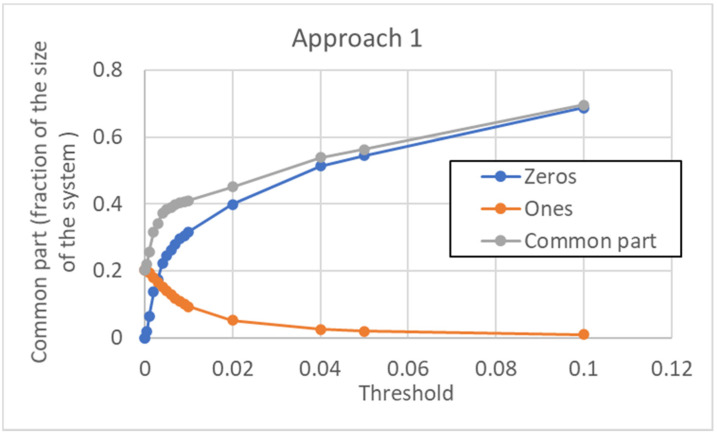
Size of the common sea vs. threshold in the case of approach 1. The CS is given by the sum of those genes that are always off (“Zeros”) in all the attractors, and of those genes that are always on (“Ones”). Note that, for very high threshold values, all the genes are ultimately switched off, the common part includes all the genes of the network, and there is only a single pseudo-attractor (0…0). In the portion of the curve that is shown here, all the pseudo-attractors are distinguishable. To have terms of comparison for the threshold values shown, the average activation value over the whole data set is 0.033, while its median is 0.0013 (the distribution of activations is skewed, with a very long right tail).

According to this approach, a unique threshold value is used for every gene of the pseudo-cell. The size of the common sea depends, of course, upon the chosen threshold, in a way that is illustrated in Figure 14. Note that the minimum size of the CS (at null threshold) is about 20%. It increases when the threshold is increased, and this is achieved by a much larger number of 0s than 1s in the type.

**Figure 15:**
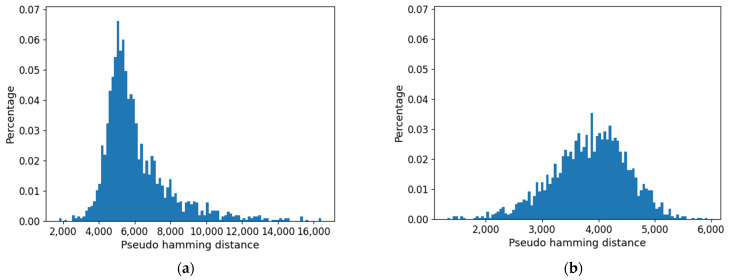
Distribution of pseudo-hamming distances between the profiles of the cell type obtained using approach 1. (**a**) Threshold = 0.0. (**b**) Threshold = 0.007.

Furthermore, the distribution of distances (which presents a maximum) is affected by the threshold: as shown in Figure 15, it is flattened when the threshold is increased. Consistently with the increase in size of the common part, the long tail of large distances tends to disappear.

### 4.2. Approach 2

According to this approach, a unique threshold value is used for every gene of the re-scaled pseudo-cell type C, as described in Section 2.5.

**Figure 16:**
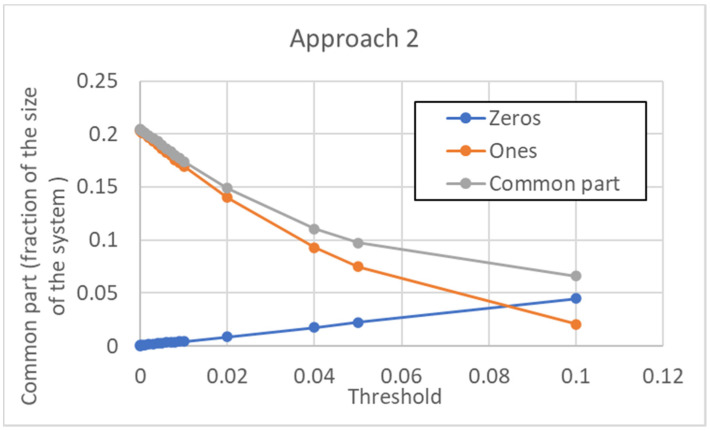
Size of the common sea vs. threshold in the case of approach 2. The CS is given by the sum of those genes that are always off (“Zeros”) in all the attractors, and of those genes that are always on (“Ones”). In the portion of the curve that is shown here, all the pseudo-types are distinguishable. To have terms of comparison for the threshold values shown, the average activation value over the whole data set is 0.11, while the median is 0.038 (the distribution of activations is skewed, with a long right tail). For larger threshold values than those shown, the number of “Zeros” is sufficient to reverse the decreasing trend of the common part, whose value begins to rise monotonically.

The dependence of the size of the common sea upon the threshold is shown in Figure 16. Note that, contrary to the previous case, in the initial part, it goes down as the threshold is increased. This shows how important the choice of the threshold can be. In this case, however, the size of the common part increases by further increasing the threshold (when the threshold becomes huge, all the pseudo-genes are, of course, not expressed).

**Figure 17:**
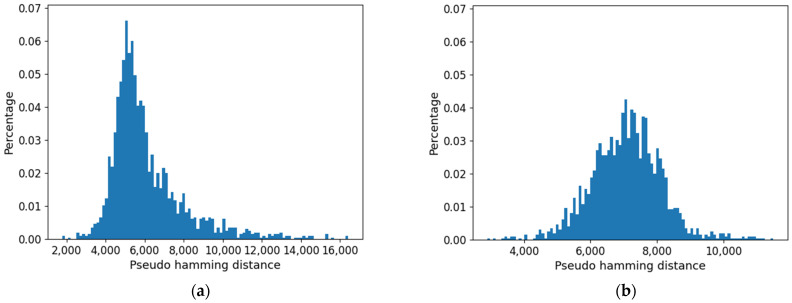
Distribution of pseudo-hamming distances between the profiles of the cell type obtained using approach 1. (**a**) Threshold = 0.0. (**b**) Threshold = 0.038.

In this case, the distribution of distances is also affected by increasing the value of the threshold (Figure 17). Consistently with the initial decrease in size of the common part, the maximum is significantly shifted to the right: it is interesting to note that also in this second approach the long tail of the large distances tends to disappear.

## 5. Conclusions

Let us recall here that the major lessons, which stem from the results described in this paper, can be summarized as follows:

1. The common sea is an important property of the RBN model;
2. Criticality does not suffice to determine the size of the CS;
3. Both the CS and the SP often host different (weakly connected) components;
4. The study of the model suggests original ways to analyze experimental data, asking novel questions;
5. The way in which real-valued activation values are binarized plays a crucial role.

### 5.1. The Common Sea Is An Important Property of the RBN Model

In order to study the behavior of the model, an important step has been the introduction of Boolean pseudo-attractors, which allows one to identify the “common sea” and the “specific islands” of the overall network. While the existence of a large fraction of “frozen” nodes, which take constant values, has been known long since, it should be stressed that the CS is a different notion, so far unexplored, i.e., that of the set of nodes that take the same value in all the pseudo-attractors but are not necessarily frozen. Indeed, in almost 80% of critical nets, the CS comprises oscillating nodes. Note also that the fraction of nodes that belong to the CS increases as the size of the network increases.

Keeping constant the overall number of nodes, one observes, as it should be expected, that the number of different attractors tends to decrease as the size of the common sea increases.

A further comment on the simulation results concerns the distribution of distances between pairs of attractors, which shows a unimodal form, initially growing, reaching a maximum for a given value of the distance, and declining after that value. The same shape is conserved even when larger networks are considered: in these cases, the maximum shifts to the right and the curve flattens.

If we abandon the condition that the network be critical, and we move towards more ordered networks (obtained, e.g., by changing the bias while keeping constant the number of nodes *N* and the connectivity *k*), then the size of the CS tends to increase, while the number of different attractors decreases.

### 5.2. Criticality Does Not Suffice to Determine the Size of the CS

Perhaps surprisingly, the same happens as the connectivity (the number of inputs per node) is increased, while keeping the network dynamically critical by a corresponding change of the bias. This is an important result: the size of the common part does not depend only upon the dynamical regime, but it differs in different ensembles of critical networks. The ordering effect due to the increased bias seems to slightly prevail on the effect of the increase in the connectivity (which, ceteris paribus, would rather tend to shift a network towards the “chaotic” region).

### 5.3. Both the CS and the SP Often Host Different (Weakly Connected) Components

The fragmentation of the CS and of the SP is extremely interesting. In critical networks with *k* = 2, only in a limited fraction of cases does the CS coincide with the whole network, while about 85% networks allow one to identify also at least one specific island.

Broadly speaking, in critical nets the CS is typically composed of a large (weakly connected) component and one or more smaller ones, while the SP is fragmented in a number of components of similar sizes (of course, these are the phenomena that are most frequently observed, while single network realizations may behave in different ways). The number of different components decreases as the size of the CS increases, and a similar phenomenon happens in the SP. The number of different attractors seems positively correlated with the size of the specific part while it does not seem to show a similar correlation with the number of subparts of the SP (data not shown). This might look surprising but it might be related to the limited size of the networks simulated so far.

The CS and the SP, and their weakly connected components, are emerging intermediate-level structures that can be used to describe the dynamical organization of the network. We suggest that looking at their properties may provide a new viewpoint to describe features of cell types, as described in the following Section 5.4.

### 5.4. The Study of the Model Suggests Original Ways to Analyze Experimental Data, Asking Novel Questions

The use of the RBN model highlighted the existence of a significant fraction of nodes in the common sea, an observation that naturally leads one to look for similar features in biological data, such as those exemplified in Section 4, which, to the best of our knowledge, had not been previously presented. It is important to stress that “playing with the model” can provide new viewpoints and can bring new questions that are useful for the study of experimental data.

While, in this paper, we analyzed some features of families of networks, note that for the notions of common sea, in specific parts, distance distribution can be applied also to specific types of organisms (a specific type of biological organism should correspond to a specific RBN). This opens the way to a number of interesting opportunities. For example, one might determine whether those genes that are always expressed in the CS coincide with the already known set of “constitutive” genes. If this is not the case, then it would be important to understand why. On the other hand, it would be interesting to check whether the set of genes that are always silent in the CS is composed of genes that are active only in particular phases of the life of the organism, such as, e.g., during embryo growth or under environmental conditions that are not represented in the sample under analysis.

In the case of unicellular organisms, where, of course, the notion of cell type cannot be applied, the different attractors are usually interpreted as different modalities of functioning; for example, when some food is lacking, or when some noxious substance is present in the environment. The very notions of CS, SP and distance distribution might be applied also in these cases.

As described in Section 3.1, it was observed that there may be different WCCs of similar size in a specific part. It might be tempting to try to understand whether the behavior of the various attractors can be approximately described by suitable combinations of the behaviors of these islands. This would be particularly simple if, for example, an island had two or a few “ways of functioning”. This approach might provide an intrinsic (i.e., not *a priori* predefined) intermediate-level description of the phase space of the system. This hypothesis is really intriguing; however, its feasibility must be confirmed by further studies of larger networks.

Last but not least, the notions of CS and SP may prove useful in comparing different organisms, if analogous lists of genes can be identified. Studying how they change during evolution would be a wonderful opportunity.

### 5.5. The Way in Which Real-Valued Activation Values Are Binarized Plays a Crucial Role

We think that virtuous feedback between theory and experimental data is necessary in biology, in a period such as the present one when there is a lack of theoretical foundations and a wealth of experimental data. We show in this paper that such a process can at least be envisaged starting from RBNs and from single-cell gene expression data, focusing on concepts such as the common sea and on the distribution of distances between cell types.

In Section 4, we showed that the size distribution of the analogue of the CS in human single-cell data depends, to a large extent, upon the choice of the threshold. In our approach 2, we also used gene-specific thresholds, albeit in a very simplified form. The threshold was formally the same for all the genes, but their range was previously rescaled to match the [0,1] interval). It will be very interesting in the future to consider some recent works [63,64], which aim at identifying the “best” threshold for binarization from the data themselves, i.e., from observed real-valued activation values (such as, for example, in [26,28]. These more sophisticated methods are very interesting and may provide quite strong constraints on the acceptable parameters of the theoretical model. However, as already remarked, a careful comparison of the model behavior with experimental data (which would require not only a more thorough analysis of the effects of binarization, but also an analysis of the behavior of RBNs with different parameter values and maybe a larger data set) lies beyond the scope of the present work; therefore, we prefer to postpone the application of these more sophisticated binarization methods to future works.

While the results presented so far are new and interesting, it is also important to acknowledge some major limitations of the present study, which represent challenges for future works. In some cases, the improvements are quite straightforward:

1. Most investigations so far concentrated on networks with *N* = 100 nodes, while different numbers could be explored (thus trying also to identify how the size of the various parts scales with *N*);
2. Different combinations of parameter values could be considered;
3. Different network random topologies could be explored (e.g., scale-free);
4. The set of Boolean functions might be limited to take into account biological plausibility [35,36,65], i.e., the possibility of being implemented in real wetware.

Other changes might require more care. A major one concerns the fact that real networks are not fully random, but have undergone long periods of evolution. If the size of the SP and its fragmentation are important from an evolutionary perspective, then they are likely to have been modified through generations. One might tentatively suggest that this explains, at least in part, the differences between the CS of simulated networks and that of single-cell data. In the end, this might lead one to question the validity of the fully random model.

Another aspect that requires great care is the effect of the external environment: RBNs are autonomous dynamical systems, while real GRNs are subject to influences from other variables (chemical species, temperature, etc.). If a Boolean network is used to model only a part of the whole human regulatory network (for example, in the networks collected in [29]), then also the other genes belong to its “environment”, and one should find effective ways to describe their effects. While there may be quite simple ways to achieve this, e.g., by clamping their values, it would still be necessary to define key concepts such as criticality in these cases.

These topics will be the subject of future works.

## Figures and Tables

**Figure A1:**
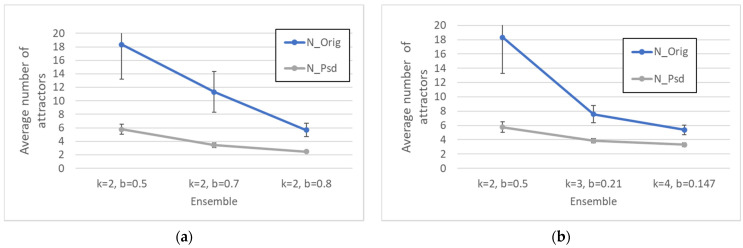
(**a**) Average number of attractors (N_Orig) and of pseudo-attractors (N_Psd) of the main ensembles with *k* = 2 used in the paper. (**b**) Average number of attractors (N_Orig) and of pseudo-attractors (N_Psd) for different types of critical networks. The error bars have a size equal to three times the standard deviation of the mean (a situation that in the case of Gaussian distributions corresponds to having more than 99.7% of the measurements inside the error bar).

## Data Availability

The tools we utilized, with comments and guides, are available at http://morespace.unimore.it/marcovillani/wp-content/uploads/sites/15/2022/02/software_entropy_2022.zip (accessed on 2 February 2022). The human DGE data we used are described in [45] and are available at https://figshare.com/articles/HCL_DGE_Data/7235471 (accessed on 2 February 2022).

## Appendix A

In order to provide a rough (mean field) estimate of the fraction of frozen genes in a very large network with *k* = 2, *b* = ½, let us consider which genes take the same value at time *t* + 1, irrespective of the values of their inputs. We can think of the extreme case of two completely random initial states, and look at the value of x at time 1, 2 etc., taking into account only the genes that are frozen (i.e., they belong to CS). The 16 two-input Boolean functions, represented in Table A1, are equally probable.

As observed in Section 3.1, the nodes with input functions f0 and f15 always take constant values. In a large random network, their fraction is likely to be 1/16 + 1/16 = 1/8. These states are frozen and equal at every time, so they belong to the first CS, call it CS1. There may, of course, be other frozen genes, but here we consider only those that are frozen (and equal for every attractor) due to their constant input functions, and in the following, we will count their frozen descendants.

**Table A1:** The 16 two-input Boolean functions.

A	B	F0	F1	F2	F3	F4	F5	F6	F7	F8	F9	F10	F11	F12	F13	F14	F15
0	0	0	0	0	0	1	0	0	1	1	1	0	0	1	1	1	1
0	1	0	0	0	1	0	0	1	0	1	0	1	1	0	1	1	1
1	0	0	0	1	0	0	1	0	0	0	1	1	1	1	0	1	1
1	1	0	1	0	0	0	1	1	1	0	0	0	1	1	1	0	1

At time *t* + 1, some nodes will be frozen because both their inputs are frozen. If at time *t*, there were 1/8 nodes frozen, the nodes with two parents that are both in CS will be 1/64, making a total of 9/64. At time *t* + 2, the nodes with both parents in CS will be (9/64)2, so the sum is

(A1) $$\frac{1}{8}+\frac{1}{64}+{\left(\frac{1}{8}\right)}^{4}+\cdots =\frac{1-{\left(\frac{1}{8}\right)}^{n}}{\frac{7}{8}}-1\;\to \;\frac{1}{7}$$

The sum of the first terms 1/8 + 1/64 is 9/64, which is only slightly smaller than 1/7. Thus, this already provides a good estimate of the fraction of nodes that are directly frozen, plus the descendants of their pairs (roughly 14%). However, this is not the whole story. Therefore, let us consider the nodes that have only one parent in CS. We will arbitrarily call A the node in CS and we will examine the Boolean functions of the downstream nodes. Consider first nodes that had been initially frozen by F0 to the value of 0. Then, the value of the downstream node whose function is *F_j_* is given here below (“-” means not frozen).

**Table A2:** The value of the 16 two-input Boolean functions, after freezing to 0 the first input (“-” means not frozen).

F0	F1	F2	F3	F4	F5	F6	F7	F8	F9	F10	F11	F12	F13	F14	F15
0	0	0	-	-	0	-	-	1	-	-	-	-	1	1	1

Let us now consider the nodes that had been initially frozen with F1, so their value is 1 (Table A3).

**Table A3:** The value of the 16 two-input Boolean functions, after freezing to 1 the first input (“-” means not frozen).

F0	F1	F2	F3	F4	F5	F6	F7	F8	F9	F10	F11	F12	F13	F14	F15
0	-	-	0	-	1	-	-	0	-	-	1	1	-	-	1

The nodes ruled by f0 and f15 have already been taken into account. Note that in both cases, six extra nodes become frozen at time *t* + 1. A fraction 1/16 of the nodes will initially (i.e., at time *t* = 1) be frozen by F0. Each such node has *k* (=2) output nodes, and each of these nodes will become frozen with probability 6/16. Then, at time *t* + 1, there will be (1/16) × 2 × (6/16) new frozen nodes. The nodes frozen by F15 follow the same rules, so the total number of newly frozen nodes at time *t* + 1 will be (2/16) × 2 × (6/16) = (2/16) × (3/4). The total number of frozen nodes will then be 2/16 + (2/16) × (3/4) frozen nodes.

At the following time step, we should study the descendants of these nodes. Each one will affect 2 × 6/16 nodes; therefore, *n*(2) = 2/16 + (2/16) × (3/4) + (2/16) × (3/4)2:

To summarize, as freezing propagates, the number of frozen nodes will be

At *t* = 0, CS(0): *n*(0) = 0;

At *t* = 1, CS(1): *n*(1) = 2/16;

At *t* = 2, CS(2): *n*(2) = 2/16 + (2/16) × (3/4);

At *t* = 3, CS(3): *n*(3) = 2/16 + (2/16) × (3/4) + (2/16) × (3/4) 2.

In general,

(A2) $$\frac{2}{16}\left[1+\frac{3}{4}+\frac{9}{16}+\cdots \right]\;\to \;\frac{1}{8}\frac{1-{\left(\frac{3}{4}\right)}^{n}}{1-\frac{3}{4}}\;\to \;\frac{1}{2}$$

therefore, as *n* grows, 50% more nodes tend to be frozen. However, this is a very rough estimate, which can either overestimate some contributions (two nodes in CS(2) may have a common descendent, and the same may happen at subsequent times, so their contributions should be subtracted) or neglect others (for example, two oscillating nodes may have a common frozen descendent). However, it suffices to provide a sound basis for the fact that the CS is large, as shown in Figure 1.

## Appendix B

The reasons for the introduction of “pseudo-attractors” are extensively discussed in the text (Section 1 and Section 2.3), and they will not be repeated here. The operation is non-injective, as it can map different dynamical attractors onto the same constant state. This Appendix shows the extent of this effect on extensive simulations of different combinations of parameter values, which are discussed in the main text.

Table A4 presents some indices characterizing the number of attractors and the number of pseudo-attractors emerging during the Booleanization procedure. The distributions of these values are very skewed so, in order to provide a synthetic characterization, we highlight that in more than 25% of the networks the number of attractors and the number of pseudo-attractors are identical (third row of the table), and that in the case of unequal numbers, their ratio is typically near to or greater than 60% (fourth row of the table).

Note also that the number of attractors and the number of pseudo-attractors have similar trends as the bias varies (with constant connectivity *k*—Figure A1a) and as the dynamic regime varies (Figure A1b).

**Table A4:** The first two rows show the average and median number of attractors of the main ensembles used in the paper (1000 networks in each ensemble), for the cases of original attractors (Orig) and pseudo-attractors (Psd). The third row indicates the fraction of networks in which the number of pseudo-attractors is equal to the number of attractors. The fourth line indicates, in the case of a change in number, the average ratio between the number of pseudo-attractors and the number of attractors.

	*k* = 2, *b* = 0.5		*k* = 2, *b* = 0.7		*k* = 2, *b* = 0.8		*k* = 3, *b* = 0.21		*k* = 4, *b* = 0.147	
	Orig	Psd	Orig	Psd	Orig	Psd	Orig	Psd	Orig	Psd
**Average**	18.34	5.77	11.32	3.44	5.67	2.49	7.58	3.85	5.38	3.31
**Median**	6	3	4	2	3	2	4	3	3	3
**Fract.unchanged**		0.24		0.24		0.29		0.36		0.39
**Fract.drop**		0.61		0.58		0.62		0.70		0.73
